# Identification of Potential miRNA–mRNA Regulatory Network Associated with Pig Growth Performance in the Pituitaries of Bama Minipigs and Landrace Pigs

**DOI:** 10.3390/ani12213058

**Published:** 2022-11-07

**Authors:** Yingying Jiao, Linlin Hao, Peijun Xia, Yunyun Cheng, Jie Song, Xi Chen, Zhaoguo Wang, Ze Ma, Shuo Zheng, Ting Chen, Ying Zhang, Hao Yu

**Affiliations:** 1 College of Animal Science, Jilin University, Changchun 130061, China; 2 Ministry of Health Key Laboratory of Radiobiology, College of Public Health, Jilin University, Changchun 130061, China; 3 Chinese National Engineering Research Center for Breeding Swine Industry, SCAU-Alltech Research Joint Alliance, Guangdong Provincial Key Lab of Agro-Animal Genomics and Molecular Breeding, College of Animal Science, South China Agricultural University, Guangzhou 510642, China

**Keywords:** miRNA–mRNA network, growth performance, pig, pituitary

## Abstract

**Simple Summary:**

The growth performance of pigs reflects their economic value. Compared with Landrace pigs, Bama minipigs have a slower growth rate and a lower feed utilization. Differentially expressed miRNA and mRNA were detected in the pituitary tissues of Bama minipigs and Landrace pigs involved in the relevant pathways mediating animal growth. In addition, we validated in vitro some potential miRNA–mRNA combinations associated with growth performance. Our findings provide an important resource for studying the differences in growth performance of different pig breeds.

**Abstract:**

Pig growth performance is one of the criteria for judging pork production and is influenced by genotype and external environmental factors such as feeding conditions. The growth performance of miniature pigs, such as Bama minipigs, differs considerably from that of the larger body size pigs, such as Landrace pigs, and can be regarded as good models in pig growth studies. In this research, we identified differentially expressed genes in the pituitary gland of Bama minipigs and Landrace pigs. Through the pathway enrichment analysis, we screened the growth-related pathways and the genes enriched in the pathways and established the protein–protein interaction network. The RNAHybrid algorithm was used to predict the interaction between differentially expressed microRNAs and differentially expressed mRNAs. Four regulatory pathways (Y-82-*ULK1/CDKN1A*, miR-4334-5p-*STAT3/PIK3R1/RPS6KA3/CAB39L*, miR-4331-*SCR/BCL2L1*, and miR-133a-3p-*BCL2L1*) were identified via quantitative real-time PCR to detect the expression and correlation of candidate miRNAs and mRNAs. In conclusion, we revealed potential miRNA–mRNA regulatory networks associated with pig growth performance in the pituitary glands of Bama minipigs and Landrace pigs, which may help to elucidate the underlying molecular mechanisms of growth differences in pigs of different body sizes.

## 1. Introduction

The growth performance of pigs is an important economic indicator, and researchers have focused on improving it by means of nutritional adjustments or changing feed ingredients [1,2]. However, growth performance is also regulated by genetic factors, which generally have a higher effect than external feed conditions [3]. The growth performances across different pig breeds vary considerably [4]. The Bama minipig is a unique Chinese miniature pig breed that is native to Bama County, Guangxi Province, presenting slight individual differences (highly inbred). Compared with the larger body size pig breeds, such as Landrace pig, Bama minipigs have a slower growth rate and a lower feed utilization efficiency [5]. Therefore, the differences between Bama minipigs and Landrace pigs are important in the research on the growth of pigs.

MicroRNAs (miRNAs), a class of evolutionarily conserved short non-coding RNAs (with a length of approximately19 to 25 nucleotides), play important roles in animal growth and cell proliferation, autophagy, and apoptosis [6]. miRNAs regulate gene expression at the post-transcriptional level by targeting the 3′ untranslated regions (3′-UTR) of mRNAs [7]. miRNAs are differentially expressed during pituitary development [8], and the reduction of miRNAs in the pituitary led to pituitary hypoplasia and growth retardation. Among them, miRNA-26b affects pituitary development by regulating the expression of the pituitary transcription factor 1 (*Pit-1*) and lymphatic enhancer factor 1 (*Lef-1*) [9]. miR-709 inhibits growth hormone releasing peptide 6 (GHRP6) induced growth hormone (GH) synthesis by targeting protein kinase C alpha (*PRKCA*) in the pituitary [10]. To our knowledge, the systematic and comprehensive analysis of the miRNA–mRNA network regulating growth performance in the pituitaries of pigs of different body sizes has not been performed.

In our previous study, we compared the expression profiles of pituitary miRNAs and mRNAs in Bama minipigs and Landrace pigs, and analyzed the miRNA–mRNA network regulating GH secretion [11]. In the present study, we further analyzed and validated the differentially expressed genes (DEGs) and differentially expressed microRNAs (DEMIRs) in the pituitaries of Bama minipigs and Landrace pigs, in an attempt to uncover the potential miRNA–mRNA networks that may be involved in regulating growth performance in pigs of different body sizes.

## 2. Materials and Methods

### 2.1. Data Source

In our previous experiments, the anterior pituitaries of three 20-day-old healthy female Bama minipigs and Landrace pigs were used for miRNA and mRNA expression profile analysis using miRNA microarrays and mRNA-seq. The miRNA microarrays data and the mRNA-seq data were submitted to the Gene Expression Omnibus (GEO) database under accession number GSE68489 (GSM1673695 and GSM1673696) and GSE68490 (GSM1673697 and GSM1673698).

### 2.2. Differential Expression Analysis and Enrichment Analysis of DEGs

Differential analysis between Bama minipigs and Landrace pigs was conducted using the DESeq2 package (http://www.bioconductor.org/packages/release/bioc/html/DESeq.html (accessed on 2 May 2020)) in R [12]. The mRNAs with the adjusted *p*-value of <0.05 and log2 fold change (FC) of >1 were selected as the DEGs. These DEGs were further utilized in the Kyoto Encyclopedia of Genes and Genomes (KEGG) [13] pathway enrichment analysis (http://www.genome.jp/kegg/ (accessed on 2 May 2020)) and Gene Set Enrichment Analysis (GSEA) [14] to expound the promising signaling pathways correlated with the overlapping DEGs. The threshold for selecting the significant results was the *p* < 0.05.

### 2.3. Protein–Protein Interaction (PPI) Network Analysis

The Metascape (http://metascape.org (accessed on 18 June 2020)) [15] and Cytoscape [16] were used to establish the PPI network. The sub-network in this PPI network was determined by applying a mature complex identification algorithm called Molecular Complex Detection (MCODE) application in Cytoscape. The genes with a degree of connectivity of >15 were defined as hub genes.

### 2.4. miRNA–mRNA Interaction Prediction

The potential miRNA–mRNA target relationships were analyzed using the RNAhybrid algorithm (https://bibiserv2.cebitec.uni-bielefeld.de/rnahybrid/submission.htm (accessed on 8 July 2020)) [17]. Briefly, we obtained all differentially expressed porcine transcripts of 3′-UTR from the NCBI database (https://www.ncbi.nlm.nih.gov// (accessed on 8 July 2020)). In the RNAhybrid algorithm analysis, the cut-off “perfect match of 2–8 seed sequence and −25 kcal/mol thermal energy, G:U matches allowed” was employed. We calculated the upregulated miRNA–downregulated mRNA to represent the proportion of negatively correlated miRNA–mRNA pairs in the RNAhybrid predicted miRNA–mRNA target pairs. Given the negative regulatory effect of miRNAs, all of the selected DEGs were downregulated mRNAs in the Bama minipigs.

### 2.5. Animal and Cell Culture

The animal experiments were approved by the Experimental Animal Center of Jilin University and conducted in accordance with the experimental practices and standards approved by the Guide for Ethical Use of Animals of the Animal Welfare and Research Ethics Committee of Jilin University (approval no. KT202003090). The pituitary cells were cultured as described in previous studies. Briefly, under sterile conditions, the pituitary glands were collected from three seven-day-old male Landrace pigs and three seven-day-old male Bama minipigs. After washing in phosphate buffer solution (PBS) three times, the posterior lobes were removed, and the anterior pituitary lobes were collected and clipped. The specimens were digested in Dulbecco’s modified Eagle medium: F-12 (DMEM/F-12) contained 0.25% trypsin-EDTA (Gibco, Grand Island, NY, USA) and 0.1 mg/mL collagenase type II (Sigma-Aldrich, St. Louis, MO, USA) for 30 min at 37 °C. Upon sufficient digestion, the emerging cell was poured through a 75 mm nylon filter (200-mesh filter) and centrifuged at 1000 rpm for 5 min. The supernatant was discarded and the resulting cells were washed with PBS (1000 rpm, 5 min) and cultured in F12K medium containing 2.5% fetal bovine serum (FBS), 15% horse serum (Solarbio, Beijing, China), and 1% penicillin/streptomycin (Gibco, Grand Island, NY, USA).

### 2.6. miRNA Transfection

All RNA oligonucleotides used in this study, including miR-133a-3p mimic, miR-4331 mimic, Y-82 mimic and negative controls, were obtained from Gene Pharma (Suzhou, China), and their sequences are shown in Table 1. The oligonucleotides were transfected using the LipoPlus™ Reagent (SageCreation, Beijing, China) according to the manufacturer’s protocol with at least three replications. The primary pituitary cells were seeded in a six-well plates for 12 h and transfected with mimics and negative controls. The cells were collected for RNA and protein extraction after 48 h.

### 2.7. Quantitative Real-Time PCR (qRT-PCR)

Total RNA was isolated from the primary pituitary cells by using RNAiso Plus (Takara, Kusatsu, Japan). The cDNAs were obtained by the PrimeScript RT Reagent Kit (Takara, Kusatsu, Japan). The miRNA reverse-transcription and the expression levels were performed by miRNA First Strand cDNA Synthesis Tailing Reaction Kit (Sangon Biotech, Shanghai, China) according to the manufacturer’s instruction. The qRT-PCR reactions were obtained using the SYBR Green PCR Master Mix (Thermo Fisher Scientific, MA, USA) with the real-time PCR Systems ABI PRISM 7900 (Applied Biosystems, MA, USA). The relative expression was normalized to the endogenous control β-actin or U6 via the 2^-ΔΔCt^ method. The primers used for qRT-PCR are listed in Table 2.

### 2.8. Statistics Analysis

All experiments were set up with three independent replicates, and the results were presented as mean ± S.E.M. Statistical analysis was performed using the GraphPad Prism Software (Version 8, La Jolla, CA, USA). Comparisons between the two groups were performed by means of Student’s *t*-tests. A *p*-value of <0.05 was considered to be statistically significant. * *p* < 0.05, ** *p* < 0.01, *** *p* < 0.001.

## 3. Results

### 3.1. Gene Set Enrichment Analysis of DEGs

**Figure 1:**
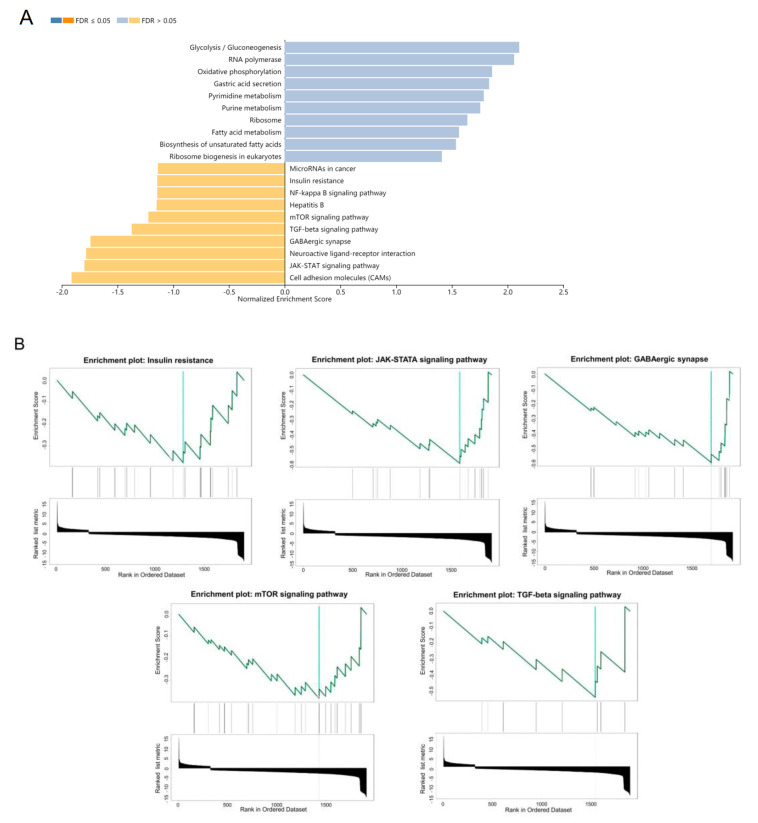
Identification of biological pathways associated with the difference in growth performance between Bama minipigs and Landrace pigs. (**A**) Top 10 enrichment results of DEGs based on the KEGG pathway database. (**B**) GSEA plot for five KEGG signaling pathways in the log2 fold change for whole transcriptome.

The DEGs analysis was conducted using the R package DEseq2. A total of 446 upregulated and 1808 downregulated DEGs of the pituitaries between Bama minipigs and Landrace pigs were identified. KEGG pathway enrichment analysis and GSEA analysis were performed to evaluate the potential biological function of DEGs (Figure 1A,B). The results showed that there were five downregulated pathways (Insulin resistance, mechanistic target of rapamycin kinase (mTOR) signaling pathway, transforming growth factor beta (TGF-beta) signaling pathway, gamma-aminobutyric acid-ergic (GABAergic) synapse, and janus kinase-signal transducers and activators of transcription (JAK-STAT) signaling pathway), which are related to cell proliferation and animal growth. At the same time, tumor pathways regulated by miRNAs were also found.

### 3.2. Construction of the Core PPI Regulation Network

**Figure 2:**
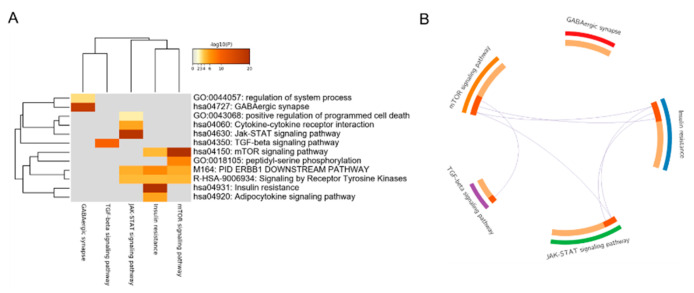
Analysis of five growth-related enrichment pathways. (**A**) Heatmap of enriched terms across enriched genes of the five pathways, colored by *p*-values. (**B**) Overlaps among the enriched genes of the five pathways at the gene level, in which purple curves link the identical genes. The inner circle represents gene lists, in which hits are arranged along the arc. Genes that hit multiple lists are colored in dark orange, whereas genes unique to a list are shown in light orange.

We further analyzed the five growth-related signaling pathways mentioned above and found that the genes in the GABAergic synapse pathway and TGF-beta signaling pathway were not enriched for biological processes in the other pathways. For the genes in the JAK-STAT signaling pathway, insulin resistance and mTOR signaling pathway shared at least two or more pathways (Figure 2A). None of the genes in the GABA pathway shared with the other four pathways (Figure 2B).

**Figure 3:**
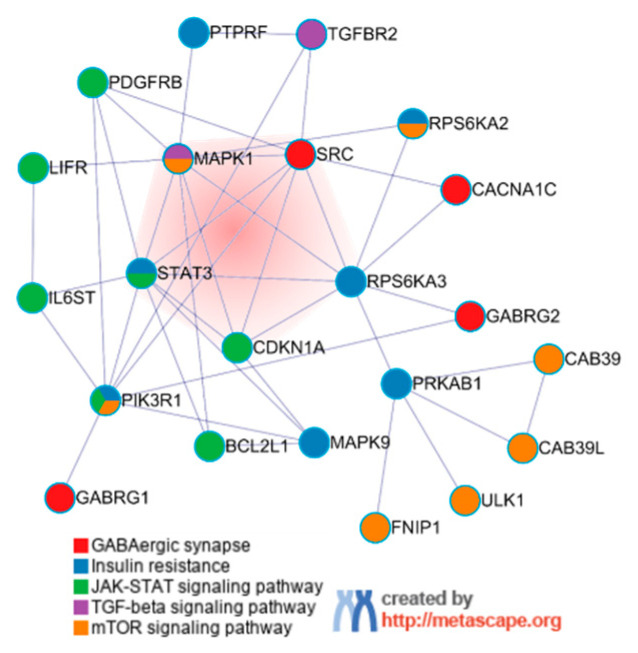
PPI network of five downregulated pathways. The red part at the center represents the MCODE components identified in the PPI network.

The core PPI network of the enriched DEGs in the five signaling pathways was generated by Metascape and visualized by Cytoscape. The network was composed of 21 nodes, including a transcription factor BCL2 like 1 (*BCL2L1*) (Figure 3). Subsequently, MCODE analysis showed only a single sub-network in the PPI network representing the core of the entire network. It consisted of *STAT3*, cyclin-dependent kinase inhibitor 1A (*CDKN1A*), ribosomal protein S6 kinase alpha-3 (*RPS6KA3*), tyrosine-protein kinase SRC proto-oncogene (*SRC*), and mitogen-activated protein kinase 9 (*MAPK9*). These genes are involved in the cell proliferation, apoptosis, and animal growth performance such as feed intake.

### 3.3. Construction of the miRNA–mRNA Interaction Network

**Figure 4:**
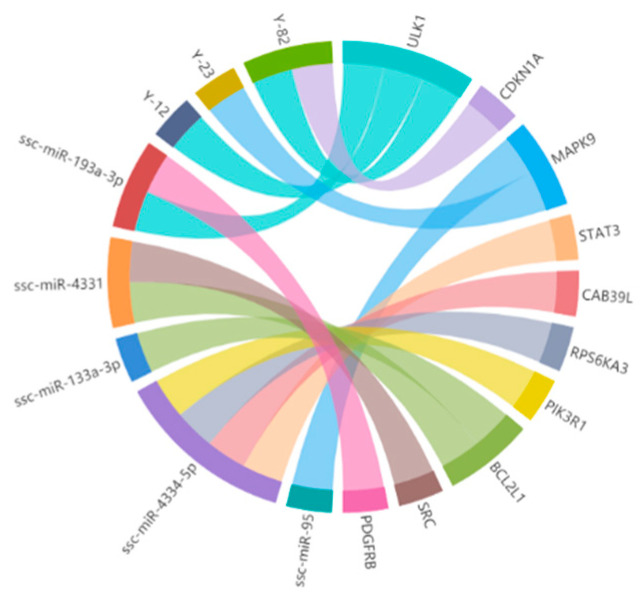
Predicted relationship of screened candidate miRNA and targeted gene of PPI network.

We have identified 41 DEMRs in the pituitaries of Bama minipigs and Landrace pigs in our previous study, including 32 upregulated and 9 downregulated DEMRs. On the basis of the local sequence alignment of the miRNA seed sequences with the 3′-UTR of the 21 nodes mentioned above and the RNAhybrid calculation, we further identified eight candidate miRNAs (Y-12, Y-23, Y-82, ssc-miR-193a-3p, ssc-miR-95, ssc-miR-4334-5p, ssc-miR-4331, and ssc-miR-133a-3p) targeting ten mRNAs (platelet derived growth factor receptor beta (*PDGFRB*)*, CDKN1A, SCR, BCL2L1,* phosphoinositide-3-kinase regulatory subunit 1 *(PI3KR1), RPS6KA3,* calcium binding protein 39 like *(CAB39L)*, *STAT3*, *MAPK9*, and unc-51 like autophagy activating kinase 1 (*ULK1*)), including five hub genes. The potential regulatory network for miRNA–mRNA interactions is shown in Figure 4.

### 3.4. Validation of Potential Interference Effects of Candidate miRNAs by qRT-PCR

**Figure 5:**
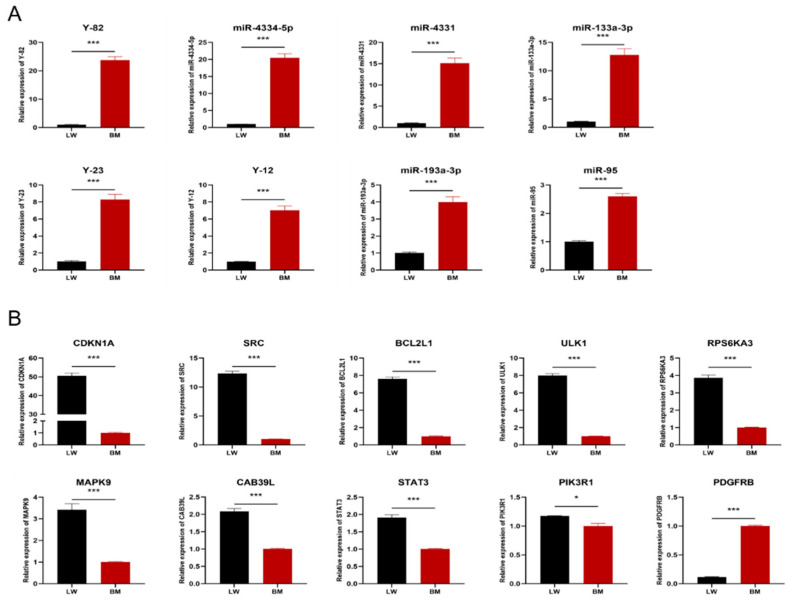
Expression levels of miRNA and mRNA in the pituitary cells of Landrace pigs and Bama minipigs. (**A**) Eight differentially expressed miRNAs. The expression abundance of Landrace pig (LW) was normalized to 1. (**B**) Ten differentially expressed mRNAs. The expression abundance of Bama minipig (BM) was normalized to 1. (* *p* < 0.05, *** *p* < 0.001.)

Aimed at validating the interaction relationship of miRNA–mRNA provided by the network, the expression levels of the DEMIRs and DEGs in the pituitary cells of Landrace pigs and Bama minipigs were assayed. As shown in Figure 5A, consistent with the results of pituitary tissue transcriptome, the expression levels of the eight DEMIRs were significantly higher in the pituitary cells of Bama minipigs than those in Landrace pigs. Furthermore, Y-82, miR-4334-5p, miR-4331, miR-133a-3p differed by more than ten-fold. Then, the expression levels of ten potential target genes were determined. The results were presented in Figure 5B. Among the ten target genes, *PDGFRB* expression in the pituitary cells of Bama minipigs was higher than that of Landrace pigs. This finding differs from the result of the bioinformatics analysis. *CDKN1A, SRC, BCL2L1, and ULK1* were expressed by more than five-fold in different levels; interestingly, they are potential target genes for Y-82, miR-4331, and miR-133a-3p.

**Figure 6:**
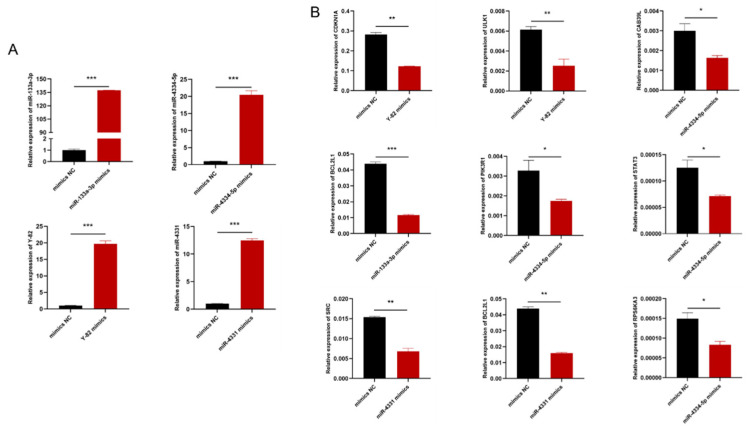
Expression levels of target genes after transfection of the potential miRNA mimics. (**A**) Efficiency of miRNA mimics after miRNA mimics transfection compared with that of the mimics negative control (NC). (**B**) The RNA level of target genes detected by qRT-PCR after the transfection of Y-82, miR-4334-5p, miR-4331, and miR-133a-3p mimics analyzed in the pituitary cell of Bama minipigs. (* *p* < 0.05, ** *p* < 0.01, *** *p* < 0.001.)

Aimed at further verifying the target relationship among Y-82, miR-4334-5p, miR-4331, and miR-133a-3p and their potential target genes, after transfection with four miRNA mimics, we found that all eight potential target genes were downregulated (Figure 6). Therefore, these findings suggest that Y-82-*ULK1/CDKN1A*, miR-4334-5p-*STAT3/PIK3R1/RPS6KA3/CAB39L*, miR-4331-*SCR/BCL2L1,* and miR-133a-3p-*BCL2L1* may be involved in regulating pig growth, leading to the differences in the growth performance between the Bama minipigs and Landrace pigs.

## 4. Discussion

The difference in growth performance between Bama minipigs and Landrace pigs is significant. According to the dietary data of National Research Council, the final body weight (BW) of Landrace pigs in the finishing phase was 92.03kg, whereas that of Bama minipigs was 52.76 kg. The average daily gain (ADG) of Landrace pigs was nearly threefold that of Bama minipigs. The average daily feed intake (ADFI) of Landrace pigs during the growing phase was slightly higher than that of Bama minipigs, and in the finishing phase, it was nearly two-fold. The feed intake to body gain ratio (F/G) of Bama minipigs was significantly higher than that of Landrace pigs by nearly 1.8-fold [18]. The pituitary gland is the most important endocrine organ regulating postnatal growth in animals [19]. The postnatal development of the pig pituitary gland may be a major factor affecting the differences in growth traits between Bama minipigs and Landrace pigs. There are very few sex differences at the mRNA levels in the pituitary gland during the neonatal period [20]. miRNA–mRNA regulatory networks have been shown to regulate multiple biological pathways and processes by means of complex relationships [21,22,23,24]. In this study, to better validate the differential expression of miRNAs and mRNAs in the pituitaries of Bama minipigs and Landrace pigs, we selected 7-day-old piglets for cellular-level validation. We have identified several miRNAs, target genes, and miRNA–mRNA regulatory pathways that may be involved in regulating the growth performance of pigs of different body sizes through a series of bioinformatic analyses and experimental validation.

In our work, four regulatory pathways (Y-82-*ULK1/CDKN1A*, miR-4334-5p-*STAT3/PIK3R1/RPS6KA3/CAB39L*, miR-4331-*SCR/BCL2L1,* and miR-133a-3p-*BCL2L1*) in the pituitary were identified to be associated with the differences in growth performance between Bama minipigs and Landrace pigs, and they may affect the growth performance of pigs by participating in the regulation of pituitary development. Among them, Y-82, miR-4334-5p, and miR-4331 are pig-specific microRNAs, but their functions have been less studied, especially the newly discovered Y-82 which has not yet been reported. One of the target genes of Y-82, *ULK1* is the core of autophagy regulation which is closely related to the regulation of appetite [25,26]. It has been found that AMP-activated protein kinase (AMPK) induced autophagy via the direct phosphorylation of *ULK1* and regulated the expression of neuropeptide Y (NPY) and pro-opiomelanocortin (POMC), thereby increasing food intake [27]. Meanwhile, the regulatory mechanism of the Y-82-*ULK1* pathway on pig ingestion requires further study. Another target gene of Y-82, *CDKN1A*, has not been reported as a growth promoter.

The expression level of miR-4331 was upregulated during mitochondrial dysfunction, and it promoted transmissible gastroenteritis virus-induced mitochondrial damage by targeting retinoblastoma 1 (*RB1*) [28,29]. Mitochondrial damage interacts with insulin resistance and promotes apoptosis [30], and it targeted *SRC* ameliorated palmitate-induced insulin resistance [31,32]. In addition, the GABA(B) receptor induced *SRC* kinase phosphorylation via insulin-like growth factor I receptor (*IGF-1R*) trans activation [33]. Bioinformatic analysis results of differential genes, including *SCR* and the GABA(A)receptor *GABRG1* and *GABRG2*, found that they are enriched in the GABAergic synapse pathway. Gamma-aminobutyric acid (GABA) is widely distributed in the peripheral endocrine organs, such as the pituitary [34]. A study conducted by Athapaththu, A. et al. found that GABA induced *IGF-1* expression through GABA(A) and GABA(B) receptors and promotes the growth rate of zebrafish larvae through the *IGF-1-IGF-1R* axis [35]. Moreover, the malfunction of signaling molecules in the *CCK/SRC/PI3K* cascade reaction with *leptin/JAK2/PI3K/STAT3* signaling pathway may lead to eating disorders [36]. We speculate that miR-4331-SCR with miR-4334-5p-*STAT3/PIK3R1* may affect the differential feed intake in Bama minipigs and Landrace pigs through the signaling pathway *CCK/SRC/PI3K-leptin/JAK2/PI3K/STAT3*. In addition, miR-4331 and miR-133a-3p jointly target *BCL2L1*, a potent inhibitor of cell death promoting cell survival [37]. Furthermore, miR-133a-3p also promoted apoptosis and inhibited cell proliferation in a variety of cell lines [38,39,40].

In miR-4334-5p-*STAT3/PIK3R1/RPS6KA3/CAB39L*: *STAT3* is a key signaling protein that triggers a variety of biological outcomes including cell growth, differentiation, and survival [41]; *PIK3R1* mediated the PI3K/AKT/mTOR signaling pathway to promote cell proliferation and inhibit apoptosis [42]; and *RPS6KA3* mediated cell proliferation, survival, and differentiation by regulating the mTOR signaling and inhibiting the pro-apoptotic functions of BCL2-associated agonist of cell death (*BAD*) and death-associated protein kinase 1 (*DAPK1*) [43]. miR-4334 has been confirmed to target Toll-like receptor 4 (*TLR4*), and the knockdown of *TLR4* inhibited the angiotensin II-induced proliferation of vascular smooth muscle cells [44,45]. Therefore, miR-4334-5p potentially inhibits the proliferative effect of cells. All the aforementioned reports hint that miR-4334-5p-*STAT3/PIK3R1/RPS6KA3* regulated the growth of pigs by mediating proliferation and apoptosis of pituitary cells. Meanwhile, *CAB39L* was considered as a promising candidate gene associated with daily feed intake and feed efficiency, but its function requires further confirmation [46].

Here, we performed a comprehensive analysis and validation of the miRNA–mRNA regulatory network associated with the differential growth performance of Bama minipigs and Landrace pigs and successfully identified several potential miRNA–mRNA pathways associated with growth in pigs. Nonetheless, we encountered limitations in this research: (1) We only selected two representative pig breeds, namely, Bama minipig and Landrace pig, to analyze the differences in growth performance of different size pigs, which has some limitations. More pig breeds should be analyzed in the subsequent studies. (2) Our study was based only on the negative correlation between miRNAs and their target mRNAs, which is restrictive. (3) The relationships between miRNAs and their targets need to be confirmed by further experiments in vitro and in vivo.

## 5. Conclusions

The preliminary experimental validation presented in this research indicates several potential miRNA–mRNA pathways contributing to pig growth performance in the anterior pituitary of Bama minipigs and Landrace pigs. The in vitro and in vivo experiments confirmed the potential negative regulatory effects of miRNAs on cell proliferation-related genes and animal feed intake-related genes in pituitary cells, especially the pig-specific miRNA, such as Y-82, miR-4334-5p, and miR-4331. The results of this research can provide a pre-study basis for exploring the effect of miRNAs on the growth differences between pigs of different body sizes.

## Figures and Tables

**Table 1:** Sequences of the miRNA oligonucleotides.

Gene	Sequences (5′–3′)
miR-4331 mimics	Sense: UGUGGCUGUGGUGUAGGCCAGC Antisense: UGGCCUACACCACAGCCACAUU
miR-133a-3p mimics	Sense: UUGGUCCCCUUCAACCAGCUG Antisense: GCUGGUUGAAGGGGACCAAUU
Y-82 mimics	Sense: UAGGGGGCAGGAGCCGGAGCCCUCU Antisense: AGGGCUCCGGCUCCUGCCCCCUAUU
NC mimics	Sense: UUCUCCGAACGUGUCACGUTT Antisense: ACGUGACACGUUCGGAGAATT

**Table 2:** Primer pairs used for qRT-PCR reactions.

Gene	Sequences (5′–3′)	Product Length (bp)	TM (°C)	Accession Number
*ULK1*	Sense: CAGTGCCGCTGTGAAGAAGT Antisense: ACCATGACAAAGTCGTCGGT	246	59	XM_021072523.1
*CDKN1A*	Sense: ACTTCGACTTCATCACTGAGACC Antisense: ACATGGTCCTCCTGAGACGT	184	59	XM_013977858.2
*MAPK9*	Sense: GACACGGTATTATCGGGCAC Antisense: TCCTCACAGTCGGCTGAAGT	212	59	XM_005661441.3
*STAT3*	Sense: ATCAAGCAGTTCCTTCAGAGC Antisense: TGGACACGCTTGCGAAC	212	59	NM_001044580.1
*CAB39L*	Sense: TCTACAATAGTGGGCTGCTGG Antisense: AGCATAATCCCACAGCGTAAG	220	59	XM_003130971.6
*PIK3R1*	Sense: TACTGTAGCCAACAACGGTATGA Antisense: CGTATTTCCCATCTCGGTGA	227	59	XM_021076847.1
*RPS6KA3*	Sense: TCTACTTGGCTGAACTTGCG Antisense: TCTGAGTGTGACCTCGACGA	228	59	XM_021080348.1
*BCL2L1*	Sense: ACTGTGCGTGGAGAGCGTAG Antisense: TCCGACTGAAGAGCGAACC	251	59	XM_021077300.1
*PDGFRB*	Sense: GTGGGCTTTCTCCCTGTTGA Antisense: GTAGGCGTCGGAATCCACTT	240	59	XM_021085013.1
*SRC*	Sense: CACCTTCCGACTCCATCCAA Antisense: TGTTGAACTGGGTGCGTGAG	252	59	XM_021077973.1
*β-actin*	Sense: TGTGCAGGGTATTCATGTGTCCGA Antisense: CAAGGCAAGTTAACAACCCACGGT	189	59	XM_021086047
miR-95	Sense: CGCGTTCAACGGGTATTTATTGAGCA	-	60	100316591
miR-4331	Sense: TATATATACCCTGGAGTGACGGGGG	-	60	100526379
miR-4334-5p	Sense: CAACTGGCCTACAAAGTCCCAGT	-	60	100526405
miR-193a-3p	Sense: TATGTGGCTGTGGTGTAGGCC	-	60	100498771
miR-133a-3p	Sense: TTGGTCCCCTTCAACCAGCTG	-	60	100316580
Y-12	Sense: TAGGGTGGAGAGATGGATGGATGG	-	60	-
Y-23	Sense: CGGAGTAGGAAGGAGGAGGGAAA	-	60	-
Y-82	Sense: TATATATAGGGGGCAGGAGCCGG	-	60	-

## Data Availability

Data are available upon a reasonable request.
